# Online Mental Health Resources in Rural Australia: Clinician Perceptions of Acceptability

**DOI:** 10.2196/jmir.2772

**Published:** 2013-09-05

**Authors:** Craig Sinclair, Kristi Holloway, Geoffrey Riley, Kirsten Auret

**Affiliations:** ^1^Rural Clinical School of Western AustraliaUniversity of Western AustraliaPerthAustralia; ^2^Curtin Health Innovation Research InstituteSchool of Nursing and MidwiferyCurtin UniversityPerthAustralia

**Keywords:** mental health, Internet, rural health, qualitative research

## Abstract

**Background:**

Online mental health resources have been proposed as an innovative means of overcoming barriers to accessing rural mental health services. However, clinicians tend to express lower satisfaction with online mental health resources than do clients.

**Objective:**

To understand rural clinicians’ attitudes towards the acceptability of online mental health resources as a treatment option in the rural context.

**Methods:**

In-depth interviews were conducted with 21 rural clinicians (general practitioners, psychologists, psychiatrists, and clinical social workers). Interviews were supplemented with rural-specific vignettes, which described clinical scenarios in which referral to online mental health resources might be considered. Symbolic interactionism was used as the theoretical framework for the study, and interview transcripts were thematically analyzed using a constant comparative method.

**Results:**

Clinicians were optimistic about the use of online mental health resources into the future, showing a preference for integration alongside existing services, and use as an adjunct rather than an alternative to traditional approaches. Key themes identified included perceptions of resources, clinician factors, client factors, and the rural and remote context. Clinicians favored resources that were user-friendly and could be integrated into their clinical practice. Barriers to use included a lack of time to explore resources, difficulty accessing training in the rural environment, and concerns about the lack of feedback from clients. Social pressure exerted within professional clinical networks contributed to a cautious approach to referring clients to online resources.

**Conclusions:**

Successful implementation of online mental health resources in the rural context requires attention to clinician perceptions of acceptability. Promotion of online mental health resources to rural clinicians should include information about resource effectiveness, enable integration with existing services, and provide opportunities for renegotiating the socially defined role of the clinician in the eHealth era.

## Introduction

Rural mental health presents a unique set of challenges in which limited resources are available to service communities already burdened by significant risk factors for mental health problems, including social isolation [1,2] and financial uncertainty [3]. Research has indicated problems for people in rural areas in accessing conventional mental health services, due to service provision shortages [4], attitudinal factors [5,6], financial and geographical barriers [7], and concerns about anonymity [7,8].

In Australia, recent developments in online technology and expanded infrastructure supporting Internet access [9] have opened the possibility for mental health services to be delivered over the Internet in rural areas [10]. In combination with a national program to upgrade access to high-speed Internet in regional areas, the Australian government has published a strategy for a nationally coordinated approach to online mental health, which includes online information resources and the development of a virtual clinic [9]. Online approaches to mental health service delivery include therapy delivered across the Internet [11], Internet support groups [12], and specific mental health websites, some of which are supported by technicians or therapists [13]. Research has established the efficacy of a number of standalone websites [14,15], and some have suggested that supplementing traditional mental health services with these online mental health resources may be effective in overcoming access issues in rural communities [10,13]. In this paper, we focus specifically on these mental health websites when using the term “online mental health resources”.

Recent studies have assessed different approaches to the delivery of online mental health resources, including community-based models [16], enhanced primary health care [17], and promotion in educational settings [18]. There is recognition that the implementation of online mental health resources in clinical settings is dependent on clinicians’ attitudes towards the resources [17,19]. Acceptability, defined as “the degree that patients, clinicians, or others are comfortable or at ease with a service and willing to use it” (p. 259 [20]), has been proposed as an important determinant of intentions to use online mental health resources. While a number of studies have demonstrated that consumers typically find online mental health resources to be acceptable [20-23], few studies have examined clinicians’ perceptions of the acceptability of online mental health resources or articulated the situations in which clinicians would consider referral of a client to online mental health resources as “acceptable”. A Norwegian study found that psychologists showed positive attitudes to therapy delivered over the Internet by a clinician, though a majority felt that this would work only as a supplement to face-to-face communication [19]. One survey of cognitive-behavioral therapists in the United Kingdom found that clinicians had low awareness of, and ambivalent attitudes toward, computerized cognitive behavioral therapy (cCBT) [24]. A systematic review of barriers to uptake of cCBT found that clinicians expressed more negative attitudes than clients [25]. Similar findings have emerged from early work in the area of online mental health resources. An Australian survey found that health professionals showed less positive attitudes toward online mental health resources than the general public and less intention to use these resources in the future [20].

While some have suggested that online mental health resources may make a significant contribution to rural mental health service delivery [10], little empirical data have been collected in rural settings [11,15]. Understanding the attitudes of rural clinicians toward the use of online mental health resources in the rural context will be important if these resources are to be widely implemented. This study adopted a qualitative approach, exploring rural clinicians’ perceptions of online mental health resources and their attitudes towards referral of clients to these resources, in the rural context.

Symbolic interactionism was employed as a theoretical framework for the study. Symbolic interactionism asserts that humans make decisions about action based on the symbolic meanings ascribed to these actions, which are learned through social interactions and reflection on the self from the imagined perspective of others [26]. In this way, a clinician’s decision about referral to online mental health resources can be thought of as a symbolic action, the meaning of which is defined based on his or her perceptions and through interactions with others. This perspective sensitizes the researcher to the contexts within which clinical decision making occurs and the importance of the social interactions that inform clinicians’ perceptions of the acceptability of online mental health resources.

## Methods

This study adopted a qualitative descriptive approach [27,28] to facilitate a thorough exploration of clinicians’ perceptions relating to online mental health resources. The method of constant comparison was used to collect and analyze the data [29]. Recruitment took place in two defined phases, and data analysis occurred concurrently with data collection. In total, 21 rural clinicians participated in individual interviews (n=17) or a group discussion (n=4), which were conducted face to face (n=19) or by telephone (n=2) and lasted for an average of 51.6 minutes (SD 15.6). A brief demographic questionnaire was administered prior to the interview, and a summary of participant characteristics is provided in Table 1.

**Table 1:** Summary of participant characteristics.

Characteristic	Phase 1	Phase 2
Age, mean (SD), years	47 (10.6)	52 (7.9)
Gender (female, male)	10 female, 3 male	3 female, 5 male
Experience providing mental health services, mean (SD), years	18.2 (9.6)	16.9 (8.1)
Remote/very remote mental health service experience, n (%)	4 (31)	7 (87.5)
General practice (n)	4	4
Mental health specialists (n)	9	4

### Phase 1

#### Sampling

In Phase 1, sampling was through formal invitations sent to community-based rural mental health organizations and convenience sampling of rural mental health specialists (psychologists, psychiatrists, and clinical social workers) and general practitioners. Two of the authors, a rural health researcher (CS) and an experienced rural psychiatrist (GR), developed an initial discussion guide in consultation with existing literature (see Multimedia Appendix 1). Open-ended questions were used to explore participants’ awareness and perceptions of online mental health resources and their typical patterns of referral of clients to online mental health resources in the rural context.

#### “Quick Guide” Resource Sheet

Anecdotal reports from clinicians during study development and previous research [24] suggested low levels of awareness of online mental health resources among clinicians. Participants were provided with a quick guide to a selection of commonly used, evidence-based online mental health resources (ReachOut [30], MoodGym [31], and BluePages [32]), as well as a portal website (Beacon [33]), all of which are based in Australia. The quick guide facilitated practitioners’ exploration of a selection of relevant websites prior to the interview, to encourage richer responses. It also encouraged participation by some who might otherwise have “self-selected” out of the study due to lack of prior experience with the resources. In this way, the present method goes some way to overcoming the self-selection bias present in a previous similar study, which recruited health professionals via the Internet [20].

### Phase 2

#### Sampling

In Phase 2, purposeful sampling recruited clinicians with experience delivering mental health services in remote or very remote settings (Accessibility Remoteness Index of Australia >5.92) [34]. The discussion guide was refined to focus on issues specific to the rural and remote context (see Multimedia Appendix 1). The interview protocol also employed a set of vignettes designed to facilitate deeper discussion and explore clinicians’ attitudes toward referral of clients to online mental health resources.

#### Vignettes

Based on data collection and analysis in Phase 1, the researchers developed a set of vignettes describing three hypothetical, rurally based clinical scenarios in which referral of a client to online mental health resources might be considered (see Multimedia Appendix 2). The clinically relevant aspects of the scenarios were standardized across interviews, but some aspects were optimized to each participant’s situation (names of towns were personalized to the participant’s catchment area, and terms such as “therapy” and “consultation” were used for mental health specialists and general practitioners respectively). Participants were asked to rate the overall plausibility of each scenario on 5-point Likert scales (1=highly implausible to 5=highly plausible) and give their opinions about the acceptability of referral to online mental health resources in each case. Eight participants recruited during Phase 2 completed the questionnaire as part of the interview protocol, and 7 participants recruited during Phase 1 were contacted again and responded by email. The mean plausibility ratings for each vignette were all greater than 4 (Vignette 1: mean 4.4, SD 1.2; Vignette 2: mean 4.6, SD 0.6; Vignette 3: mean 4.7, SD 0.6).

### Analysis

Interview transcripts were analyzed using NVivo Version 10, following an iterative process of open, focused, and theoretical coding to extract codes, categories, and themes from the interview transcripts. Relational coding was used to articulate relationships between open codes and to identify categories [35]. Two rurally based health researchers (CS, KH), with training in psychology and nursing respectively, independently coded the first 10 transcripts and discussed any discrepancies until consensus. This led to the development of a preliminary coding framework. During ongoing data analysis (conducted by CS), an audit trail documented addition of any new codes or changes to the coding framework. Discussion of the themes among the research team, which included an experienced rural psychiatrist (GR), ensured the clinical relevance of the findings reported.

Rigor of the research process is demonstrated by its credibility, dependability, confirmability, and transferability [36]. Credibility was addressed by involving the interviewer throughout the analysis process. Dependability was enhanced by a thorough methodological process and clear audit trail. To address confirmability, exemplars are provided that illustrate key themes, and member checking was incorporated during Phase 2 [37]. The inclusion of health professionals from various mental health disciplines and locations of practice helps to establish the transferability of findings of this study.

The Human Research Ethics Committees of the University of Western Australia (RA/4/1/4660) and the Western Australian Country Health Service (2012:01) approved the study protocols.

## Results

### Overview and Key Themes

Clinicians framed their responses using examples drawn from their experiences delivering mental health services in the rural context. The key themes extracted from the transcripts were “perceptions of resources”, “clinician factors”, “client factors”, and “the rural and remote context”. An overarching theme of “integration with existing services” characterized participant responses.

### Perceptions of Resources

Clinicians typically expressed positive perceptions towards online mental health resources and perceived that client use was on the increase. The perceived effectiveness of online mental health resources was attributed primarily to the provision of clear and easily accessible psychoeducation, which could be used in early intervention, helping clients normalize symptoms and encouraging future help-seeking. Most acknowledged that they received limited feedback from clients who were using the resources, precluding direct comments about clinical efficacy. Some clinicians had identified their own “favorite” resources, which they referred to regularly and integrated into their clinical practice, often by printing handouts for clients. Clinicians emphasized that resources providing clear, quickly accessible information would be most appropriate for clients:

I mean one of the things I noticed is if you get to a home page and there are too many options and go here, go there, and all these things hanging off, it can be quite overwhelming and thinking about some of the minds and emotional states that my clients might be in and how this is going to feel for them.

Psychologist

An emphasis on “usability” had the added benefit of enabling clinicians to integrate the resources within the time demands of their clinical practice, allowing them to guide clients through an online resource or quickly access printable information. Others suggested that user-friendly online resources could be made available on public computers in clinic waiting rooms, assisting in psychoeducation, or providing material for further discussion with the clinician.

### Clinician Factors

Clinicians preferred to be familiar with online mental health resources prior to recommending them to clients. However, they experienced difficulties finding time to explore the range of available resources:

The brief look I have had has been done on the run…with a specific client in mind just to see what was there…it hasn’t been done with sufficient time and focus to really seriously engage and immerse myself with what is there…

Psychologist

Clinicians who were younger and trained more recently tended to show acceptance for the integration of online approaches within their everyday practice:

We’re being trained and informed about these online resources. The older doctors, I don’t imagine, are using them as much.

General Practitioner Registrar

Older, more experienced clinicians sometimes reported barriers associated with computer literacy, as well as a more general lack of exposure to online mental health resources during their training. For rural clinicians, this was compounded by the difficulties associated with accessing ongoing professional development. Some participants who had received professional development associated with a particular online mental health resource then felt more comfortable integrating it into their routine practice. Clinicians who were unable to access professional development looked to their professional networks for guidance, with mixed results. In one case, a clinician told of receiving criticism from some colleagues for raising a question about online approaches to mental health service delivery:

I recently asked…whether anyone had had any experience [using online mental health resources] among our group and everyone said, “No” and kind of suggested that even to consider that seemed ridiculous.

Psychologist

The benefits and concerns relating to online mental health resources showed some patterns of variation across professional groups. Mental health specialists (psychologists, psychiatrists, clinical social workers) were more likely than general practitioners to identify the provision of psychoeducation and early intervention as benefits. However mental health specialists also emphasized the importance of an ongoing therapeutic relationship as a mechanism for client recovery and were concerned that unsupervised use of online mental health resources might encourage self-diagnosis and “catastrophizing”.

You can get people getting online and then getting into the information online and if you were anxious and a worrier before you started wait ‘til you have spent a couple of hours online looking at how anxious and worried you really could be, so I think there can be a bit of a snowball, some catastrophizing can occur.

Clinical Social Worker

General practitioners, on the other hand, tended to identify the capacity for clients to access psychoeducation in their own time as a benefit. Both groups endorsed the ability of online mental health resources to enable greater access to mental health services, but were concerned about the lack of ability to follow up with clients about their progress.

### Client Factors

Participants identified a range of client factors thought to influence suitability for referral to online mental health resources. Younger people (particularly adolescents) were perceived as having greater computer literacy and being more willing to seek information online. Those with common mental disorders such as anxiety or depression, and symptoms in the mild to moderate range, were also considered to be more suitable than clients with complex diagnoses or severe and persistent symptoms:

Any [condition] that sort of begs the question about psychoeducation, you know what can people find out themselves, how can they gather knowledge quickly and effectively themselves.

Clinical Social Worker

Clients who were prone to excessive rumination or who lacked the motivation or attention to read information online were considered less suitable. Participants also expressed concern that some clients in rural areas lacked private Internet access, had reading difficulties, or lower levels of computer literacy.

### Rural and Remote Context

Clinicians consistently identified the rural and remote context as one in which people had less access to mental health services, and less choice among service providers. They reported that concerns about anonymity in small communities left many rural clients unwilling to access specialist mental health services. Online mental health resources provided an opportunity for rural clients to access information confidentially, and clinicians endorsed use of the resources where they might assist in normalizing symptoms and encouraging future help seeking. On the other hand, some clinicians identified the potential for certain rural environments to be a recipe for social isolation and rumination and saw the potential for online mental health resources to have a negative impact, particularly if used as a sole source of information:

In certain particularly remote environments that might then intensify the focus on you know what comes through this [online] medium.

Psychologist

Clinicians reported that many rural clients lacked reliable Internet access or sufficient privacy to access online mental health resources confidentially. While regional centers were relatively well serviced, access was less consistent in more remote areas, and the lack of community resources marginalized poorer people. One clinician felt that this constituted an area of rural disadvantage:

Often online services have been looked at as, you know, the great hope for areas where there aren’t real services for people, but where there isn’t adequate Internet access, you are further marginalizing people who live in remote areas who now don’t have access to two different services…

Psychiatrist

Some clinicians who expressed concerns about rural disadvantage feared that a reallocation of investment toward online mental health resources might compromise the provision of adequate community mental health services in rural areas.

### Integration With Existing Services

In describing their current referral practices or responding to the hypothetical vignette scenarios, clinicians considered the interaction of clinician, client, and resource factors within the rural and remote context. Their responses were characterized by a preference for integration of online mental health resources alongside existing services. Clinicians acknowledged that referral came with the risk of negative outcomes, including the client feeling neglected, experiencing frustration due to poor Internet access or lack of computer literacy, or misinterpreting online information. Some foresaw that this could lead to a loss of trust in the ongoing relationship, perhaps resulting in disengagement, along with an escalation of symptoms, for which they felt personally responsible:

You send someone off to a machine and they kill themselves or their child or something. It would be very hard to live with, wouldn’t it?

General Practitioner

Acknowledgment of these risks contributed to a preference for online mental health resources to be used as an adjunct, rather than an alternative, to face-to-face therapy. Clinicians managed the client’s use of online mental health resources, fostering realistic expectations, and using online information as material to further develop their therapeutic relationship with the client:

So what I am trying to do there is…try and manage it so they have a good experience, you know, a positive experience, then they will keep using it, but if they have that sort of frustrating adverse [experience]…then they’ll often overgeneralize and just disengage.

Psychologist

Referral decisions tended to be more polarized when the client experienced unwillingness or severe difficulty in accessing face-to-face services. Some clinicians feared that the use of online mental health resources in this situation might lead clients to disengage entirely from face-to-face services. Others suggested online mental health resources to clients who they felt were likely to disengage, in the hope of establishing a “bridge” for future contact. One clinician felt that the vignette describing “Matthew” (see Multimedia Appendix 2) was a situation where online mental health resources might be used to good effect to maintain a relationship with the client:

If you have the feeling that [client] is not going to come back again, because he can’t be forced, it’s not a decision of mine, it’s a decision of his. Like that [online personality questionnaire] is actually very useful for them to come back…there’s actually material that enables you to provide some more face-to-face work.

Psychiatrist

In some cases, the potential to refer clients to online mental health resources appeared to have changed the nature of the clinical relationship. Some clinicians referred clients to online mental health resources as a means of strengthening the therapeutic relationship. The “prescription” of homework validated the client’s concern, and access to a second opinion enabled clients to evaluate the clinician’s diagnosis and be more in control of their condition:

People then have permission to become a little bit expert themselves about whatever their condition is.

Clinical Social Worker

However, not all clinicians viewed this change in a positive way. One general practitioner referred to clients bringing information from online mental health resources to consultations to support their argument that they did not require medication:

I had a couple of young patients who very clearly were people that you would want to consider medication for, who actually quoted that site as evidence that, yeah, it didn’t have a role.

General Practitioner

## Discussion

### Principal Findings

This paper addresses a gap identified in previous literature [11,15] reporting on the perceptions of clinicians towards online mental health resources, and their use of these resources, in the rural context. The barriers to accessing mental health services in the rural setting have been well documented [4-7,38,39]. Participants in this study were optimistic about the use of online mental health resources as an innovative means of overcoming some of the barriers to accessing rural mental health services, supporting previous literature [10,13]. However, concerns that referral to online mental health resources may have adverse effects in some situations contributed to a preference that online approaches were employed as an adjunct rather than an alternative to traditional forms of mental health service delivery. This finding is consistent with previous literature, which has shown that clinicians typically express less positive attitudes towards online mental health resources than do clients [20,25,40].

Rural clinicians showed a preference for integrating online approaches with existing services. For example, the preference that online mental health resources present clear, simple, and quickly accessible information was motivated by concerns for the client but also by a desire to integrate these resources within the time demands of clinical consultations. Others suggested that providing public computers in clinic waiting rooms could be helpful. In both cases, usability was a key factor. The “technology acceptance” model proposes that the perceived usability and perceived usefulness of a technological aid will determine the extent to which it is accepted and adopted [41]. This model has been cited in previous literature relating to consumer acceptance of online mental health resources [42]; it may also be relevant in understanding acceptance and adoption by clinicians. However, it should also be noted that perceptions of “usability” are contextual and may differ between clinicians, clients, and website developers [40].

Researchers employing symbolic interactionism in the study of online behavior in nonclinical contexts have suggested that while transition to an online medium may “revolutionize” the surface features of social interaction, the underlying attributes of human action and interaction remain stable [43]. Robinson et al found that, after an initial process of redefining social rules in the online environment, the types of social interaction deployed online were characterized by an augmentation of existing practices, or “evolution”, as opposed to “revolution” [43]. When considering referral of clients to online mental health resources, clinicians preferred to see them used as an adjunct rather than an alternative to existing services. When they did refer clients to online mental health resources, it was typically an attempt to strengthen or enrich an ongoing clinical relationship. Within the framework of symbolic interactionism, clinicians’ preference for a carefully managed augmentation of existing services can be interpreted with reference to the socially defined role of the clinician and the symbolic significance of the act of referral. Our observation of social pressure exerted by some clinicians on their colleagues to reject online approaches illustrates how tension can arise when a treatment clashes with this socially defined role. From this perspective, clinicians incorporate intrinsic judgments of acceptability, along with the expectations accompanying their socially defined role, in responding to the clinical, ethical, and social implications of referral to online mental health resources. The socially defined expectations as to how mental health service delivery proceeds (client seeks help by reporting symptoms to a clinician, who establishes a relationship and delivers face-to-face therapy) may constitute a barrier to the implementation of online mental health resources in partnership with clinicians. However, research has shown how role expectations can respond to cultural shifts. One study found that general practitioners reported increasing numbers of patients bringing health information from the Internet to consultations and that some responded by restructuring their role from “gatekeeper to secondary care to facilitator of information interpretation and decision-making” (p. 93 [44]). A recent study showed that referral by a clinician to an online mental health resource resulted in higher levels of participation when the method of referral maximized the client’s intrinsic motivation, through provision of patient-focused information, rather than reliance on clinician recommendation [45]. Interviews with clients using online mental health resources have illustrated the important ongoing role of the clinician as a provider of support and facilitator of deeper understanding of information accessed through online mental health resources [46]. Changing the socially defined role of the clinician to that of a facilitator of patient-directed information seeking and decision making may be an important precursor to more effective referral of clients to online mental health resources, resulting in greater uptake and reduced attrition.

It is important to recognize that the clinician’s decision to refer to online mental health resources occurs from within a pre-existing clinical relationship. Research into the mechanisms of successful psychotherapy has stressed the importance of the clinical relationship or “therapeutic alliance” between the clinician and client [47]. Rodin and Janis theorized that the clinician’s ability to encourage client adherence to clinical recommendations relies on the clinician’s possession of “expert” power (possession of valuable information and skills) and “referent” power (social influence stemming from a strong and trusting clinical relationship) [48]. Clinicians acknowledged and endorsed the expert power of evidence-based online mental health resources to provide high quality psychoeducation. However, their concerns about the resources being used as an alternative to traditional face-to-face therapy may stem from the absence of referent power in the online environment or the potential for referral to online mental health resources to have a negative impact on the clinician’s referent power. Concerns about negative impacts of referral to online mental health resources on the clinical relationship (eg, loss of trust, client feeling neglected) may partly explain the difficulties experienced in previous attempts to implement online mental health service delivery through referrals in primary health care settings [17].

### Limitations

The convenience sampling method used in the present study is a limitation. It is possible that the sampling method may have led to a self-selecting bias, in which clinicians who were particularly opposed to the use of online mental health resources decided not to participate. The combination of data from a group discussion alongside that collected from individual interviews also adds a further layer of complexity to the analysis. However, we observed similar thematic content in both group and individual interview settings.

Another limitation is associated with the small number of public sector (government-employed) mental health professionals recruited. These professionals typically see a greater proportion of clients with severe and persistent mental illness and may be more sensitive to the risks of adverse consequences. Despite this limitation, those public sector employees who did participate were able to provide insight into the differences experienced in this context and the implications for the use of online mental health resources.

The use of vignette scenarios to frame discussions about referral to online mental health resources is useful for eliciting factors affecting decision making but does not enable reliable inferences to be drawn about future behavioral intentions in similar situations [49]. However, the high plausibility ratings given by participants suggests that the vignette scenarios provided a useful foundation for more in-depth discussion about the specific factors influencing referral decisions in a number of concrete scenarios relevant to the rural context.

### Implications

The referral of a client by their clinician is just one of a number of ways in which a client may discover and access online mental health resources. Research in Australia has mirrored trends across the developed world, showing that the public are increasingly using the Internet as a source of information about health conditions [22,50]. As health services evolve to meet changing societal expectations, it can be expected that rates of clinicians referring clients to online mental health resources will also increase. Further research is needed to explore the perceptions of health professionals in other rural and remote areas and the educational needs of clinicians relating to the use of online resources.

The argument that clinicians negotiate both intrinsic judgments of acceptability and the expectations accompanying their socially defined role, when making decisions about referral to online mental health resources, has a number of clinical and educational implications. First, it suggests that efforts to promote online mental health resources should target professional networks, as well as individual clinicians, using collaborative in-service approaches to address educational requirements and encourage cultural change.

The technology acceptance model suggests that both perceived usefulness and perceived usability will contribute to adoption of the technology [40]. This suggests that promotion of online mental health resource “usefulness” (by communicating results of clinical trials) should not be at the expense of the more basic enabling work, which underlies the perception of “usability” within the everyday clinical environment.

### Conclusion

The impressive results yielded by some trials of online approaches to mental health service delivery have led some commentators to call for “disruptive innovation” to improve outcomes [51]. The present research suggests that in the rural context, clinicians favor a more conservative approach, in which online approaches augment traditional face-to-face approaches to mental health service delivery. Ongoing negotiation of the clinician’s role in the emerging eHealth era will be a crucial factor in enabling widespread integration and implementation of online mental health resources.

## Multimedia Appendix 1

Discussion guides used in Phases One and Two.

## Multimedia Appendix 2

Vignettes used during Phase Two.

## Abbreviations

cCBT: computerized cognitive behavioral therapy
