# Supplementary material for: Online Mental Health Resources in Rural Australia: Clinician Perceptions of Acceptability
Source: J Med Internet Res. 2013 Sep 5;15(9):e193. doi: 10.2196/jmir.2772 (PMC3785998; doi:10.2196/jmir.2772)
Supplement: Supplementary file 1 [file jmir_v15i9e193_app1.pdf]

Multimedia Appendix 1. Discussion Guide – Phase One.

1. Before becoming involved in this research what was your awareness of online mental health resources?
2. Do you currently recommend online mental health resources to your clients?
    a. if yes –
        i. which resources?
        ii. why?
        iii. which types of patients?
        iv. how do clients respond?
    b. if no –
        i. why not?
        ii. what would need to change for you to consider this?
3. Do clients come to you asking about online mental health resources?
4. How effective are the currently available online mental health resources? (capable of clinically significant symptom reduction in relevant populations)
5. How acceptable are the currently available online mental health resources? (acceptable to clients and clinicians)
6. How usable are the currently available online mental health resources? (convenient and well matched with workplace demand)
7. How do you feel about the use of online mental health resources in the rural context?
8. What would be the main benefits of using online mental health resources in the rural context?
9. What would be your main concerns about using online mental health resources in the rural context?

Discussion Guide – Phase Two

1. Before becoming involved in this research, what was your awareness of online mental health resources?
2. Can you tell me about your current use of online mental health resources?
    a. Which resources do you use?
    b. Which types of patients?
    c. Why do you feel these people are particularly suitable?
    d. How do clients respond to these resources?
3. Do clients come to you asking about online mental health resources?
4. How effective are the currently available online mental health resources?
    a. Tell me what you think contributes to effectiveness in online mental health resources?
5. How acceptable are the currently available online mental health resources?
    a. Tell me what you think contributes to acceptability in online mental health resources?
6. How usable are the currently available online mental health resources?
    a. Tell me what you think contributes to usability in online mental health resources?

7. How do you feel about the use of online mental health resources in the rural context?
    a. in rural centers/towns?
    b. in remote/very remote areas?
8. Tell me how you imagine online mental health resources being used in the rural and remote context into the future?

Is there anything that would encourage you to utilize online mental health resources in your current work?
