# Supplementary material for: Online Mental Health Resources in Rural Australia: Clinician Perceptions of Acceptability
Source: J Med Internet Res. 2013 Sep 5;15(9):e193. doi: 10.2196/jmir.2772 (PMC3785998; doi:10.2196/jmir.2772)
Supplement: Supplementary file 2 [file jmir_v15i9e193_app2.pdf]

**Multimedia Appendix 2.** Vignette 1: "Matthew".

Matthew is a 16-year-old male, who lives in [Albany/Esperance/Kalgoorlie] and attends a local high school. He broke up with his girlfriend six months ago, and was referred by a school counselor after showing continuing behavioral problems and declining performance at school. He has recently been diagnosed with depression (adjustment disorder) and has attended twice in the last month for [consultation/therapy], at the request of his parents. He says he would prefer not to come, as he is worried that his friends will find out, and says that he doesn't want to be labeled 'crazy'. He has been having trouble sleeping, and recently told you that he has been 'googling' for information about depression.

Vignette 2: "Amanda"

Amanda is a 34-year-old female, who lives in [Gnowangerup/Salmon Gums]. She has recently given birth to her second child and reports feeling lethargic and flat. She recently made a large number of online purchases, which have led to arguments with her husband and feelings of distress. Both she and her husband are considering relationship counseling, but have found it difficult to access childcare services to enable them to attend.

Vignette 3: "Greg"

Greg is a 56 year old male who is married and owns a farm 20 kilometers outside of [Ongerup/Ravensthorpe]. He has been having trouble sleeping and complains of worries about his financial situation. Recently he has been drinking more alcohol at night in an effort to get to sleep. He has brought the results of an online anxiety scale, which he recently completed and scored "highly anxious". He is in debt and says that he can't afford to spend time away from the farm to come for [therapy/consultation]. He has had digestive complaints for the last three months, which ease when he has time off work.
